# Real-time motion artifact suppression using convolution neural networks with penalty in fNIRS

**DOI:** 10.3389/fnins.2024.1432138

**Published:** 2024-08-06

**Authors:** Ruisen Huang, Keum-Shik Hong, Shi-Chun Bao, Fei Gao

**Affiliations:** ^1^Shenzhen Institute of Advanced Technology, Chinese Academy of Sciences, Shenzhen, Guangdong, China; ^2^Institute of Future, Qingdao University, Qingdao, Shandong, China; ^3^School of Mechanical Engineering, Pusan National University, Busan, Republic of Korea; ^4^National Innovation Center for Advanced Medical Devices, Shenzhen, Guangdong, China

**Keywords:** neural networks, functional near-infrared spectroscopy, artifact rejection, wavelet, balloon model

## Abstract

**Introduction:**

Removing motion artifacts (MAs) from functional near-infrared spectroscopy (fNIRS) signals is crucial in practical applications, but a standard procedure is not available yet. Artificial neural networks have found applications in diverse domains, such as voice and image processing, while their utility in signal processing remains limited.

**Method:**

In this work, we introduce an innovative neural network-based approach for online fNIRS signals processing, tailored to individual subjects and requiring minimal prior experimental data. Specifically, this approach employs one-dimensional convolutional neural networks with a penalty network (1DCNNwP), incorporating a moving window and an input data augmentation procedure. In the training process, the neural network is fed with simulated data derived from the balloon model for simulation validation and semi-simulated data for experimental validation, respectively.

**Results:**

Visual validation underscores 1DCNNwP’s capacity to effectively suppress MAs. Quantitative analysis reveals a remarkable improvement in signal-to-noise ratio by over 11.08 dB, surpassing the existing methods, including the spline-interpolation, wavelet-based, temporal derivative distribution repair with a 1 s moving window, and spline Savitzky-Goaly methods. Contrast-to-noise ratio (CNR) analysis further demonstrated 1DCNNwP’s ability to restore or enhance CNRs for motionless signals. In the experiments of eight subjects, our method significantly outperformed the other approaches (except offline TDDR, *t* < −3.82, *p* < 0.01). With an average signal processing time of 0.53 ms per sample, 1DCNNwP exhibited strong potential for real-time fNIRS data processing.

**Discussion:**

This novel univariate approach for fNIRS signal processing presents a promising avenue that requires minimal prior experimental data and adapts seamlessly to varying experimental paradigms.

## 1 Introduction

Functional near-infrared spectroscopy (fNIRS) is an emerging technique that enables portable brain monitoring and long-term cerebral imaging (Cohen, 1973; Kumar et al., 2017; Lopez-Larraz et al., 2018; Hong and Yaqub, 2019; Quaresima and Ferrari, 2019; Hong et al., 2020; Alzahab et al., 2021; Khan et al., 2021; Wang et al., 2021). Some studies have claimed that artifacts caused by subjects’ or users’ movement could hamper data decoding and degrade device performance (Scholkmann et al., 2014; Naseer and Hong, 2015; Vitorio et al., 2017). Studies on the motion artifact (MA) removal began in 2003, when Izzetoglu et al. (2005, 2010) first proposed an adaptive filter. Some studies have shown that MAs stem from diverse sources. Facial muscle movements (Izzetoglu et al., 2005; Yucel et al., 2014a,b), body movements (Rea et al., 2014; Dybvik and Steinert, 2021), and movements of the subjects’ head, including nodding, shaking, and tilting (Radhakrishnan et al., 2009; Robertson et al., 2010; Kim et al., 2011; Gökçay et al., 2019) can introduce MAs to measured fNIRS signals.

Several solutions have been proposed that incorporate additional hardware to either detect subjects’ movements during experiments or fix the optodes on the subject’s scalp. Some studies have incorporated accelerometers to actively track MAs (Kim et al., 2011). Virtanen et al. (2011) presented an accelerometer-based motion artifact removal (ABAMAR) algorithm for an offline analysis. They claimed that introducing an accelerometer could improve the detection of baseline MAs. In addition, collodion-fixed prism-based optical fibers have been used to avoid relative movement between optodes and the scalp (Yucel et al., 2014b). Moreover, a pair of polarized films were used to attenuate optode-fluctuation-induced MAs (Yamada et al., 2015). In practical applications, hardware-based solutions may increase the overall system cost and are typically suited only to specific scenarios. Consequently, exploring alternative methods for motion artifact removal in more universal applications becomes essential.

The removal of MAs can be achieved using algorithmic solutions. These solutions can be classified as univariate and multivariate methods based on whether the methods use only a single signal or more signals (Scholkmann et al., 2014). Some multivariate methods utilize spatial information from different signals to determine the absolute value of tissue oxygen saturation (StO2) (Matcher et al., 1997; Hueber et al., 1999). Additionally, the principal component analysis can be used based on the spatial filtering of eigenvectors (Zhang et al., 2005; Selb et al., 2015).

Many univariate MA removal methods have been presented. Wiener and Kalman filters require prior experiments to determine parameters in their models (Izzetoglu et al., 2005, 2010; Seghouane and Ferrari, 2019). Spline interpolation, also called the movement artifact removal algorithm (MARA), is another offline filtering approach integrated into some open-source fNIRS data processing toolboxes (Scholkmann et al., 2010). Cui et al. (2010) proposed a correlation-based signal improvement method based on the assumption of a negative correlation between oxyhemoglobin (HbO) and deoxyhemoglobin (HbR) concentrations. They also proposed a correlation-based signal improvement method (CBSI). Moreover, we proposed a dual-stage median filter (DSMF) to target online filtering (Huang et al., 2022). Other methods include the wavelet-based method (Molavi and Dumont, 2012; Liang et al., 2021), temporal derivative distribution repair (Fishburn et al., 2019), and transient artifact reduction algorithm (Selesnick et al., 2014).

The domain of signal processing for fNIRS data, specifically in the context of motion artifact removal, has yet to fully leverage the potential of neural network-based methodologies. Despite the demonstrated success of artificial neural networks in diverse fields (Yang et al., 2020; Chen et al., 2021; Qing et al., 2021; Parada-Mayorga et al., 2023), their application in fNIRS signal processing remains scarce. Despite the existing conventional solutions, we propose a novel neural network-based method to address the critical issue of motion artifact removal in fNIRS signals. Section 2 provides an in-depth exploration of the proposed method’s architecture, highlighting its unique design features. In Section 3, we delve into the intricacies of our training strategy, the experimental setup, and the evaluation procedures employed to validate our approach. Sections 4 presents the results of our validation process, encompassing simulated and experimental data. In both cases, we compare the performance of our proposed method with the conventional spline-interpolation method, wavelet-based method, temporal derivative distribution repair (TDDR) method (Fishburn et al., 2019), and the spline Savitzky–Golay (spline SG) method (Jahani et al., 2018). Lastly, in Sections 5 and 6, we offer a comprehensive discussion and conclude the manuscript, shedding light on the method’s significance and potential applications in fNIRS signal processing.

## 2 Convolution neural networks with a penalty network

### 2.1 Structure

**Figure 1:**
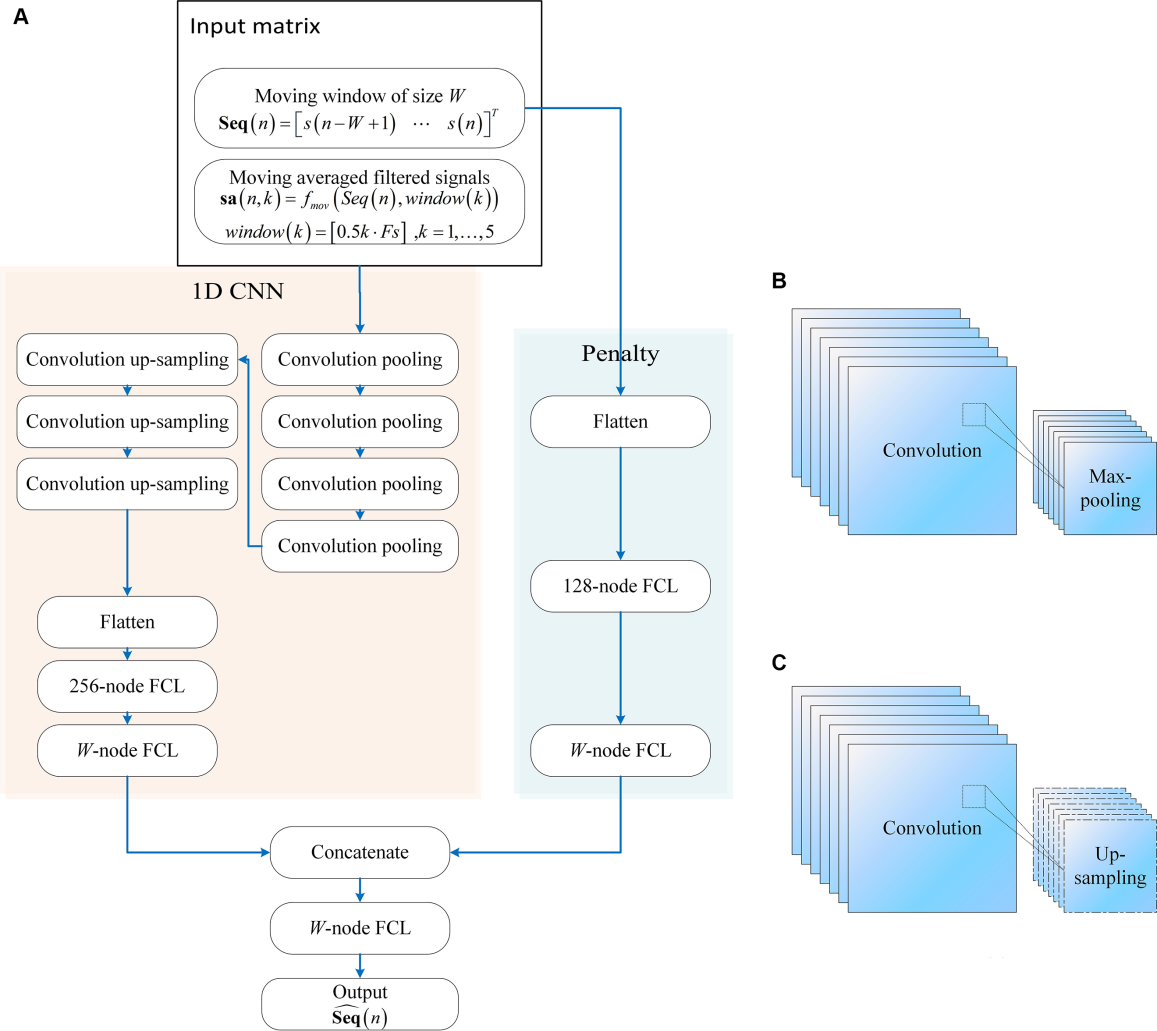
The structure of the proposed convolution neural networks with penalty: **(A)** 1D CNN consists of convolution pooling block and convolution up-sampling block, the convolution pooling block is shown in **(B)**, and the convolution up-sampling block is shown in **(C)**. FCL denotes fully-connected layer.

The proposed neural network comprises two main components as in Figure 1: A one-dimensional convolutional neural network (1D CNN) and a penalty network. The 1D CNN, adept at capturing temporal patterns in fNIRS signals, includes seven convolutional layers. The first four layers are followed by max-pooling layers, while the subsequent three layers are followed by up-sampling layers. The final up-sampling layer’s outputs are flattened and processed through a 256-node fully connected layer (FCL), with an output layer matching the input moving window size. The penalty network operates on the same inputs, flattening the data and processing it through a 128-node FCL, followed by an output layer matching the moving window size. The outputs from both networks are concatenated and passed through an FCL to produce the final network output. This synergy between the 1D CNN and the penalty network enhances robustness and efficiency. The model construction scripts are available at: https://gitee.com/cognoholic/1-dcnnw-p.git.

The design of the 1DCNNwP structure stems from a strategic integration of convolutional processes and a penalty network to address specific challenges in motion artifact removal. The choice of a CNN is driven by its ability to enhance information flow among neurons efficiently while significantly reducing the number of parameters compared to fully connected networks. However, the inherent limitations of CNNs in ensuring robust training necessitate the introduction of a penalty network operating in parallel. This auxiliary network’s output size aligns with that of the CNN, with each element in the penalty network’s output serving as a weight for the corresponding element in the CNN’s output. This unique structural configuration enhances the network’s robustness by introducing an additional layer of complexity, mitigating the risk of overfitting, and contributing to the overall stability of the training process (Zhang et al., 2014, 2021). When applied to MAs removal, the penalty network serves as an additional layer of complexity that enhances the network’s robustness by operating in parallel with the CNN. It can help by assigning weights to the CNN’s output, allowing the network to focus on the most relevant features while suppressing the less important ones. This can improve the accuracy of motion artifact removal, ensuring that the final output is free from artifacts and contains only the true underlying neural signals.

The penalty network is designed as a three-layer fully connected neural network. The first layer takes the same input as the 1D CNN, which is flattened and processed through a 128-node FCL. This is followed by a second FCL layer, also with 128 nodes, introducing an additional layer of complexity and aiding in feature extraction. The final output layer of the penalty network matches the moving window size, providing weights that act as a regularization mechanism. This structure enhances the network’s ability to generalize and improves its robustness by focusing on the most relevant features while mitigating overfitting. The penalty network’s outputs are concatenated with those of the 1D CNN, ensuring the final predictions are refined and accurate.

### 2.2 Inputs

The inputs are meticulously curated to encompass both temporal context and augmented information. In Eq. 1, for any given measured data, *s*, a fixed time window of 8 s is utilized to capture a sequence of data points, **Seq**, corresponding to the target time instant, *n*:


(1) $$\text{Seq}\left(n\right)={\left[s\left(n-W+1\right)\;\;\ldots \;\;s\left(n\right)\right]}^{T}$$

(2) W=[8⋅Fs]

In Eq. 2, *W* is the window size corresponding to 8 s and *Fs* is the sampling frequency of the data. The operator [·] denote the function to return the closest integer to the given value. To better identify and mitigate motion artifacts, the extracted data sequence is augmented by incorporating additional signals, **sa**(*n*, *k*). As shown in Eq. 3, these supplementary signals are obtained by applying a series of moving average filters with varying window sizes (0.5 s, 1 s, 1.5 s, 2 s, and 2.5 s) to the original data sequence:


(3) $$sa\left(n,k\right)=f_{mov}\left(\text{Seq}\left(n\right),\text{window}\left(k\right)\right)$$

(4) window(k)=[0.5k⋅Fs]

In Eq. 4, the variable, *window*(*k*), marks the window size of the *k*th moving average filter. The function, *f_mov_*(·,·), returns the moving average of the given signal at the given window size. The resulting filtered signals are then integrated into the original data sequence, effectively augmenting it to a matrix with six columns. The values in the matrix are normalized to the average and the standard deviation of the column afterwards. The final augmented matrix, with each row representing a time window and the columns corresponding to the original data and the additional signals (*W* × 6), forms the input data of a single wavelength for both the 1D CNN and the penalty network.

This augmentation strategy serves three purposes: The incorporation of signals filtered at different temporal scales enhances the network’s ability to capture diverse patterns present in the data. This is particularly relevant for capturing motion artifacts with varying durations. By including signals smoothed with moving average filters, the network becomes more robust to high-frequency noise and transient fluctuations in the raw data. Augmenting the original data sequence with filtered signals accounts for potential signal delays and fluctuations, offering a more comprehensive representation of the temporal context.

### 2.3 Loss functions and metrics

The design of the loss functions aims to minimize two key components: the discrepancy between the estimated motionless data sequence and the ground-truth motionless data sequence, and the error between the predicted signal at the target time instance and the corresponding true motionless signal at that time instance. Specifically, the loss function (Eq. 5) is the weighted sum of the mean-squared error (MSE) of the estimated data sequence and the ground-truth motionless data sequence (Eq. 6), and the MSE of the signal at the target time instance (Eq. 7):


(5) $$\text{Loss}\left(n\right)=w_{1}\cdot L_{1}\left(n\right)+w_{2}\cdot L_{2}\left(n\right)$$

(6) $$L_{1}\left(n\right)=\frac{{\left(\hat{Seq}\left(n\right)-Seq_{\text{true}}\left(n\right)\right)}^{2}}{W}$$

(7) $$L_{2}\left(n\right)=\frac{{\left(\hat{s}\left(n\right)-s_{\text{true}}\left(n\right)\right)}^{2}}{W}$$

Eq. 5 encourages the network to learn representations that align with the true underlying data characteristics, ensuring accurate reconstruction of the original data sequence. Eq. 6 enforces the network to focus on capturing the temporal dynamics and artifacts at the specific time point of interest. The optimization process adjusts the network’s parameters to minimize these MSE components, promoting accurate motion artifact removal.

The design of the loss functions for the proposed motion artifact removal algorithm incorporates crucial considerations for real-time applications and effective signal tracking. To address the time delay associated with a non-overlapping moving window sampling strategy, which could adversely impact real-time performance, we opted for an overlap of *W* - 1 for a moving window of size *W*. This choice aims to ensure that the estimation of the latest data point remains less susceptible to motion artifacts. The loss function (5) is strategically crafted to facilitate the accurate tracking of the signal pattern in motionless signals, ensuring that the algorithm maintains fidelity to the underlying physiological signals. Additionally, the inclusion of loss function (6) serves to fine-tune the algorithm’s parameters, minimizing estimation errors and enhancing the precision of motion artifact removal. This multi-faceted approach in the design of loss functions aligns with the overarching goal of achieving robust, real-time motion artifact removal while preserving the integrity of the underlying signals.

## 3 Training data and experimental design

### 3.1 Simulation

The training data were simulated according to the following procedures: (i) A paradigm comprising a 10-s rest for both baseline and interval between tasks. Eight tasks with durations as 5 s to 19 s (stepping 2 s) were designed to simulate the tasks with different durations. The tasks and pauses were expressed in a binary array (ones for tasks and zeros for rest). (ii) Normalized hemodynamic responses were computed using a balloon model (Buxton et al., 1998, 2004; Zheng et al., 2005). (iii) Physiological noises, including cardiac and respiratory noises, was simulated using zero-phase sinusoidal signals. Simulated physiological noises were linearly added to hemodynamic signals. (iv) Hemodynamic signals were converted into optical densities using the modified Beer–Lambert law for two wavelengths (*λ*_1_: 690 nm and *λ*_2_: 830 nm). (v) The generated optical densities were converted to optical intensities for both wavelengths using a selected reference optical density. (vi) MAs were modeled and added to the simulated optical intensities after convolution with a random binary paradigm.

Simulated signals were constructed using a balloon model and mBLL (Essenpreis et al., 1993; Huang and Hong, 2021). The balloon model was established using the neurovascular coupling model (Zheng et al., 2002, 2005; Huang et al., 2022). The model enables the simulation of normalized hemodynamic responses of any set paradigm and provides results for both HbO and HbR concentration changes. Physiological noises of random magnitudes were added to the generated hemodynamic signals and with mBLL, with the hemodynamic signals were converted to optical intensities, to which MAs of random magnitudes were added subsequently. Random magnitudes of MAs and physiological noises enhanced the training dataset such that the trained NNs were more robust to noise magnitudes. The proposed filter was trained using simulated data and semi-simulated data for real data filtering; this approach was considered equivalent to model-based reasoning (Le et al., 2017). The semi-simulated data were obtained by augmenting real baseline measurements at a simulated paradigm with varying task durations with synthetically generated motion artifacts. The process emulates different patterns of motion artifacts that can occur in practical fNIRS measurements, offering a more realistic training ground for signal processing algorithms and providing a controlled environment for evaluating an algorithm’s robustness without the cost and time associated with collecting extensive new experimental data.

Two types of MAs were considered: (i) one had a spike shape, and (ii) the other had a step shape. As shown in Eqs. 8–10, normalized spike-shape MAs were described using a unity triangular function lasting for 2*t_d_*, where *t_d_* is the desired duration of normalized artifact (here, we assign it as 1 s by default). Subsequently, spike-shape MAs were finalized by convoluting the normalized spike-shape MAs with a random paradigm, mathematically expressed as follows (Huang et al., 2022):


(8) $$TR\left(t\right)=\{\begin{array}{cc} 1+t/t_{d} & -t_{d}\leq t\leq 0,\\ 1-t/t_{d} & 0<t\leq t_{d},\\ 0 & \text{otherwise}, \end{array}$$

(9) $$MA_{\text{sp}}=TR\left(t\right)*\text{Paradig}m_{\text{sp}},$$

(10) $$\text{Paradig}m_{sp}=\{\begin{array}{cc} 1 & \text{if spike-like}\;MAs\;\text{start},\\ 0 & \text{otherwise}. \end{array}$$

Similarly, as shown in Eqs. 11–13, the step-shape MAs were modeled by convoluting a ramp function lasting for *t_d_* with a random paradigm of the artifacts:


(11) $$\text{Ramp}\left(t\right)=\{\begin{array}{cc} 0 & t\leq 0,\\ t/t_{d} & 0<t\leq t_{d},\\ 1 & \text{otherwise}, \end{array}$$

(12) $$MA_{\text{st}}=\text{Ramp}\left(t\right)*\text{Paradig}m_{\text{st}}$$

(13) $$\text{Paradig}m_{\text{st}}=\{\begin{array}{cc} 1 & \text{if step-like}\;\text{MAs}\;\text{start},\\ 0 & \text{otherwise}. \end{array}$$

Step- and spike-shape MAs were linearly added to the simulated optical intensities obtained in Step 5, forming the final synthesized optical intensities corrupted by MAs. The 1 s in Eqs. 9–12 is a sample value. In practice, the duration of the motion artifacts was modified according to different needs.

Ten sets of training data were obtained using this procedure. Each set was generated using a randomly assigned magnitude of physiological noise and MAs.

### 3.2 Experimental design

**Figure 2:**
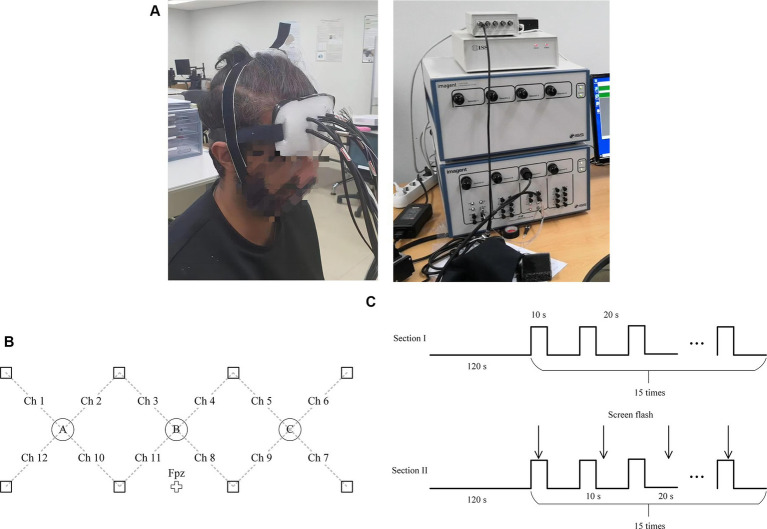
**(A)** A subject was doing experiment (left) and the ISS Imagent (right). **(B)** Configuration of fNIRS channels. Squares and circles indicate emitters and detectors, respectively. **(C)** The experiment paradigm was consisted of two sections.

The frequency-domain fNIRS system (ISS Imagent from ISS Inc., U.S.) was used to acquire optical data from the prefrontal cortices of eight subjects (two females and six males, 26.9 ± 2.75 yr). All subjects had no reported history of mental disorders and all have normal eyesight or corrected-to-normal. A channel was defined for every detector-laser pair in the neighborhood, yielding 12 channels for each subject. The detector-laser distance for each channel was set to 3.0 cm. The optode configuration is illustrated in Figure 2.

Before the experiments, the subjects provided written consent comprising all details of the experiments. Sequentially, they became familiar with the tasks by completing printed warm-up exercises (24 three-digit additions or subtractions). In the first session of experiments, the subjects were provided with a rest for 120 s at the beginning, followed by 15 repetitions of 10-s mental arithmetic tasks, which was followed by a rest for 20 s. In the second the session of the experiment, the subjects were asked to redo the experiment. In this session, the subjects were asked to shake their heads randomly when the screen flashed within each trial (total 15 × 8 = 120 times) as shown in Figure 2C. All experiments were performed in accordance with the latest Declaration of Helsinki (Christie, 2000). The experiment has been approved by the Institutional Review Board (IRB) of the Shenzhen Institute of Advanced Technology, Chinese Academy of Sciences, under the approval code SIAT-IRB-231215-H0702.

### 3.3 Training, optimization, and testing

For the model designed to handle simulation data, the training process commenced with an initial learning rate of 1.0 × 10^−4^. The model underwent training for a total of 120 epochs, with a batch size of 64. To assess the model’s generalization capability, a 5-fold validation strategy was adopted.

At the end of each fold’s training phase, a dynamic learning rate adjustment was implemented. If the validation losses at the current fold were smaller than the previously recorded best loss, the initial learning rate was halved. Additionally, during the training process, the learning rate was automatically halved every 20 epochs. This strategy facilitated fine-tuning the learning rate to achieve optimal convergence while preventing overshooting in optimization.

For models designed to process experimental data, a transfer learning approach was adopted. The model parameters obtained from the simulation data-trained model served as the foundation for training on experimental data. This approach aimed to leverage the knowledge gained from the simulation data and adapt it to real-world scenarios.

The training process for experimental data closely mirrored that of simulation data, with a few modifications. The semi-simulated data was utilized for training. They were obtained by extending baseline signals from 3 to 15 s in the experiments to form a series of 9 trials, each interspersed with 10 s of rest. Within these extended signals, spike-like and step-like motion artifacts of varied magnitudes and durations were linearly introduced at 8-s intervals. This method was applied across two wavelengths for each channel of all eight subjects, resulting in a total of 192 distinct sets (2 wavelengths × 12 channels × 8 subjects) of data. The inclusion of semi-simulated data in the training process aimed to enhance the model’s real-world applicability by exposing it to more representative scenarios, thereby enhancing the model’s ability to generalize across different types of disturbances encountered in fNIRS signals.

Similar to simulation data training, an initial learning rate of 1.0 × 10^−4^ and a batch size of 64 were employed. However, an early-stop mechanism was introduced. If no further improvement in validation loss was observed, the training for the current fold was halted, and the model proceeded to the next fold.

The utilization of transfer learning allowed for the efficient adaptation of the model’s parameters to experimental data, optimizing performance and convergence speed. The early-stop mechanism ensured that the training process did not stagnate, enhancing the model’s robustness and generalization to new data.

The testing data used in this study was derived from 192 sets of experimental recordings, each exceeding 19 min in duration. These data were utilized for testing purposes, with participants instructed to randomly shake their heads during each trial, either during a task or rest period. This ensured a minimum of 120 motion artifact occurrences for testing, while also potentially increasing the number of unique motion artifact patterns based on wavelength, channel, and subject.

In short, the training datasets for the study were formulated through a combination of simulated (for simulation study) and semi-simulated (for experimental study) data, encompassing a diverse range of task durations and motion artifacts. These sets were rigorously partitioned to facilitate a 5-fold cross-validation process, iteratively employing four subsets for training and one for validation, ensuring comprehensive model training. For the simulated data study, the testing dataset included novel motion artifacts at various positions and magnitudes. Concurrently, the testing dataset used in the experimental study was derived from actual measurements across eight subjects.

### 3.4 Evaluation

We assessed filter performance from two aspects: (i) suppressing MAs and (ii) distorting the original signal. When verifying the proposed method on the simulated data, we computed the difference in signal-to-noise ratios (SNRs) (Eq. 14) to assess the artifact suppression performance, with the difference in correlation coefficients (Eq. 15), used to assess the signal distortion (Izzetoglu et al., 2005):


(14) $$\begin{array}{l} \Delta SNR=SNR_{e}-SNR_{i}\\ =10\cdot \log_{10}\left(\frac{\mathrm{var}\left(s_{0}\right)}{\mathrm{var}\left(s_{0}-\hat{s}\right)}\right)-10\cdot \log_{10}\left(\frac{\mathrm{var}\left(s_{0}\right)}{\mathrm{var}\left(s_{0}-s\right)}\right), \end{array}$$

(15) $$\Delta CC=\text{corr}\left(s_{0},\hat{s}\right)-\text{corr}\left(s_{0},s-s_{0}\right)$$

The variable *s*_0_ denotes the motionless signals, a hat over *s* denotes the estimated signals, and *s* denotes the original signals. The function var.(·) outputs the variance of the input signals, and the function corr(·, ·) outputs the correlation coefficient of the two signals.

As motionless signals were unavailable for the experimental data, studies have tried either using real data with known hemodynamic responses and motion artifacts (Brigadoi et al., 2014) or using resting-state fNIRS data augmented with synthetic artifacts and hemodynamic responses (Von Luhmann et al., 2020). Due to the experimental design, this study will utilize reference signals from a session without motion artifacts (Session I) as a surrogate ground truth for evaluating the performance of filtering methods on data from a session with intentionally introduced motion artifacts (Session II). The first-, second-, and infinite-norm differences (*d*_1_, *d*_2_, and *d*_∞_) were used to quantify the absolute error, root-mean-squared error, and errors in extremum between the filtered signals and the reference signals, providing a comprehensive evaluation of the filtering methods’ ability to recover the underlying neural signals in the presence of motion artifacts (Chu and Delp, 1989; Sun et al., 2002). The contents of Session I were designed to be nearly identical to those of Session II, with the key difference being the absence of motion artifacts due to head shaking. This controlled design enables us to compare the signals from both sessions effectively. The reference signals from Session I, after simple smoothing, were used as the basis for calculating the metrics *d*_1_, *d*_2_, and *d*_∞_. These metrics quantify the absolute error, root-mean-squared error, and errors in extremum, respectively, between the filtered signals from Session II and the reference signals. When using *d*_1_, *d*_2_, and *d*_∞_ to compare the measured and filtered signals, the smaller the metrics, the higher the performance of the proposed method.

## 4 Results

**Figure 3:**
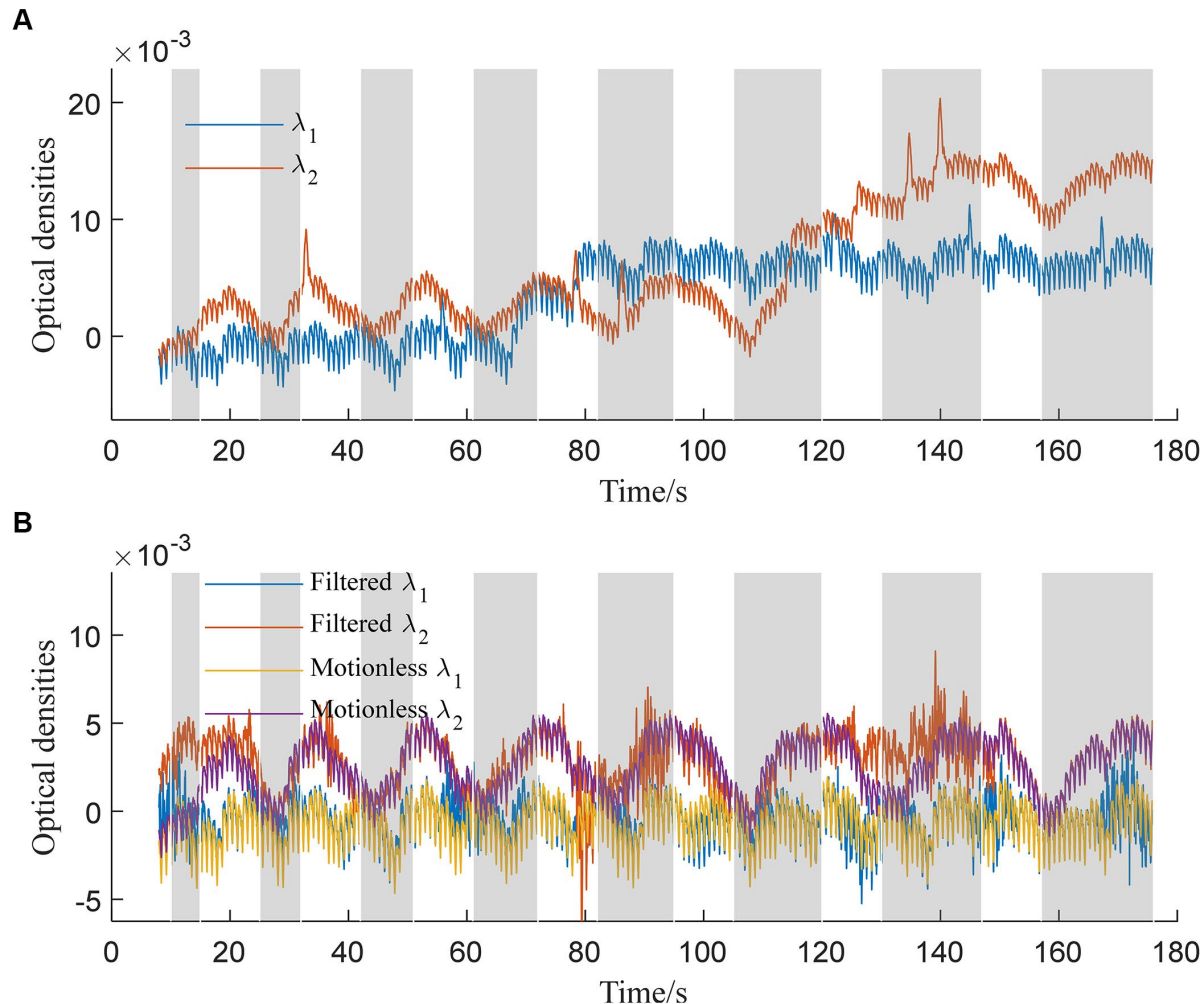
Simulated optical densities: **(A)** With motion artifacts, **(B)** without motion artifacts and the filtered signals for both wavelengths *λ*_1_ and *λ*_2_.

The trained 1DCNNwP filter was first tested using the simulated data. The simulated and filtered data are shown in Figure 3. Figure 3A illustrates the optical densities of simulated data corresponding to wavelengths *λ*_1_ and *λ*_2_. Task periods are denoted by gray color bars, while motion artifacts are marked at 33 s, 86 s, 113 s, 125 s, 134 s, and 140 s for *λ*_2_ and at 55 s, 65 s, 78 s, 145 s, and 170 s for *λ*_1_. Artifacts at 65 s, 78 s, 113 s, and 125 s are distinctly recognizable as deviations from the baseline optical densities. In contrast, the remaining artifacts predominantly manifested as spike-like anomalies. In Figure 3B, the behavior of filtered signals is depicted. Initial bias is observed, followed by a close alignment with motionless signals from 25 s onwards. Importantly, the updated results indicate successful suppression of artifacts occurring at 33 s, 78 s, 86 s, 113 s, 125 s, 134 s, and 145 s. However, some over-corrections are noticeable at 78 s, 86 s, and 170 s. The proximity of motion artifacts between 125 s and 140 s adds complexity to artifact removal, resulting in slight distortions in the filtered signals during this interval.

**Figure 4:**
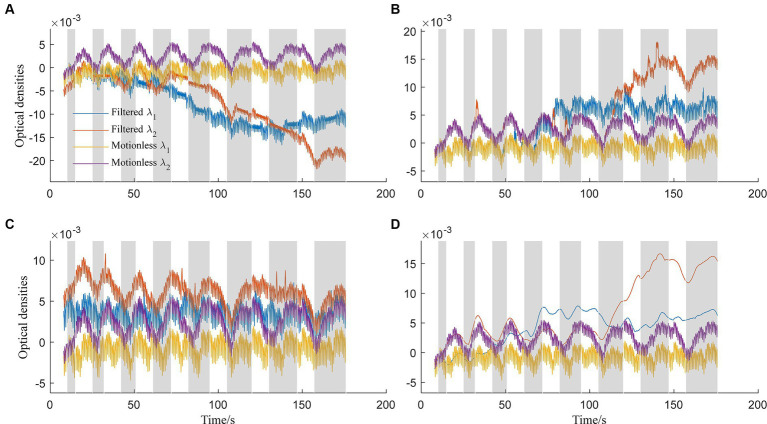
Optical densities filtered by: **(A)** Spline interpolation method, **(B)** wavelet-based method, **(C)** TDDR method, and **(D)** spline SG method for both wavelengths *λ*_1_ and *λ*_2_.

In contrast, Figure 4A displays optical densities filtered using spline interpolation (standard deviation threshold: 5, amplitude threshold: 0.1, p: 0.997). Blue lines represent *λ*_1_, and orange lines represent *λ*_2_, while yellow and purple lines depict ground-truth motionless signals. However, all three methods, including the spline interpolation method, face challenges in effectively removing baseline drifts. Notably, all methods except for the TDDR struggle with baseline drifts. The deviation between filtered signals at *λ*_2_ and ground-truth signals is attributed to sensitivity to hyperparameters. Figure 4B illustrates the application of the wavelet-based method (Haar wavelet, possibility threshold: 0.0005, window size: 2 s) for artifact removal. This method demonstrates competence in suppressing spike-like artifacts effectively. However, it may encounter difficulties in scenarios involving physiological noises or higher-frequency noise components. Moreover, it lacks a specific correction mechanism for step-like artifacts. The filtering results using the TDDR filter, as demonstrated in Figure 4C, highlight the filter’s effectiveness in maintaining stable baselines, unlike the other three methods evaluated. However, the results also reveal the TDDR filter’s limited capability in removing spike-like motion artifacts. While it excels at preserving the integrity of the baseline signal, its performance in eliminating short, sharp disturbances is comparatively constrained. Figure 4D explores performance of the spline SG (p: 0.99, frame: 10 s) filter (Jahani et al., 2018). The filter shares a common limitation with the spline interpolation filter, particularly in its tendency to induce baseline shifts in the presence of consecutive step-like artifacts. This observation suggests that while the filter is effective in smoothing the signal, it struggles to maintain the baseline integrity during prolonged or repeated disturbances. Additionally, the high-frequency components of the signals were suppressed by the filter.

Comparing our proposed method (Figure 3) to existing approaches (Figure 4), it’s evident that the latter faces distinct challenges. Spline interpolation (Figure 4A) exhibits sensitivity to hyperparameters, affecting baseline drift. The wavelet-based method (Figure 4B) excels in suppressing spike-like artifacts but struggles with step-like noises. The TDDR method (Figure 4C) excels in baseline correction but face difficulty in suppressing spike-like artifacts because these artifacts may consist some high frequency components, which are skipped by TDDR. The spline SG (Figure 4D) is capable of smoothing out rapid fluctuations while potentially compromising the detection of subtle, fast-changing physiological signals and well suppressing spike-like artifacts. Subsequently, all filters find it difficult to deal with consecutive step-like artifacts. This underscores the significance of training and dataset quality for our method. Achieving stability and minimizing over-corrections necessitates meticulous training, hyperparameter tuning, and real-world dataset representation. This comparison highlights the method’s potential for robust artifact removal, emphasizing the importance of a well-structured training process and dataset.

Quantitatively, the proposed method was further evaluated by considering ΔSNR and ΔCC; A large ΔSNR indicates effective artifact suppression, and a significant ΔCC suggests less signal distortion. Table 1 offers a comparative analysis of the performance of various filtering methods in terms of ΔSNR and ΔCC, crucial metrics for evaluating artifact suppression efficacy and signal fidelity. Among the evaluated filters, the offline TDDR filtering method exhibits the most remarkable performance, achieving the highest ΔSNR values (16.06 for *λ*_1_ and 13.16 for *λ*_2_) and ΔCC values (0.76 for *λ*_1_ and 0.64 for *λ*_2_), thus indicating robust artifact suppression with minimal signal distortion. However, a variant of the TDDR method with a 1-s moving window without overlap, referred to as TDDR (1 s), showed significantly reduced effectiveness, with ΔSNR values dropping to 7.15 for *λ*_1_ and 7.17 for *λ*_2_, and ΔCC values to 0.47 for *λ*_1_ and 0.40 for *λ*_2_, highlighting the impact of windowing strategy on filtering performance, revealing a compromise between artifact suppression and signal fidelity.

**Table 1:** ΔSNR and ΔCC of the noncorrected simulated fNIRS signals and outputs of different filters.

	ΔSNR of *λ*_1_	ΔSNR of *λ*_2_	ΔCC of *λ*_1_	ΔCC of *λ*_2_
Non-corrected	0	0	0.30	0.30
MARA	6.28	11.60	0.37	0.55
Wavelet	7.13	7.28	0.42	0.40
TDDR (offline)	16.06	13.16	0.76	0.64
TDDR (1 s)	7.15	7.17	0.47	0.40
Spline SG	6.50	6.64	0.10	0.25
1DCNNwP	11.08	11.47	0.69	0.55

Among the methods compared, the proposed 1DCNNwP, which is applicable for real-time filtering, shows commendable performance. At *λ*_1_, it surpasses all filters except for the offline TDDR, with a ΔSNR value of 11.08 and a ΔCC of 0.69, indicating a good balance between noise suppression and signal preservation. At *λ*_2_, 1DCNNwP achieves the second-highest ΔSNR (11.47), closely trailing the MARA filter (11.60). Both 1DCNNwP and MARA tie in ΔCC (0.55), offering better signal preservation than other methods aside from the offline TDDR. These results underscore 1DCNNwP’s effectiveness in real-time applications, offering a balance between noise reduction and preservation of the original signal characteristics, a vital consideration for practical implementations.

**Figure 5:**
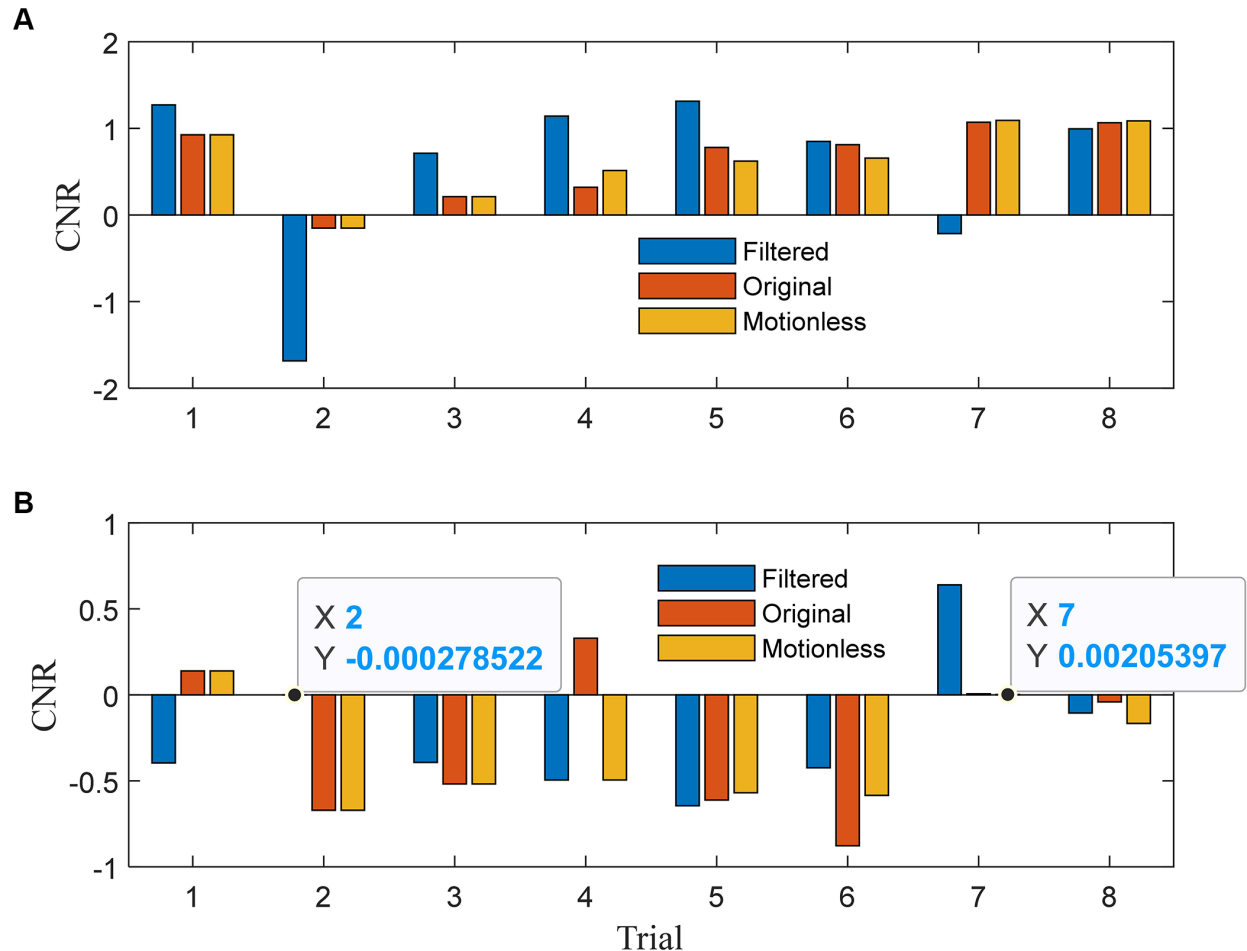
Contrast-to-noise ratio of **(A)** *λ*_1_ and **(B)** *λ*_2_ for each trial.

In Figure 5A, we present the contrast-to-noise ratios (CNRs) for HbOs. Our expectation was that the CNRs for the filtered signals would exhibit similar signs to those of the CNRs for the motionless signals. For HbOs, the filtered signals predominantly achieved CNRs that aligned with the motionless signals across all eight trials, except for the 7th trial. Moreover, in six out of seven aligned trials, the absolute CNRs for the filtered signals were greater. This observation underscores the efficacy of our method in preserving CNR alignment with motionless signals and enhancing the absolute CNR values in most cases. Moving to Figure 5B, we present the CNR results for HbRs. In seven out of eight trials, the CNRs of the filtered signals closely mirrored those of the motionless signals. Any misalignment in the first trial could be attributed to transient effects at the beginning. Notably, in the fourth trial, the original signal exhibited a positive CNR due to motion artifact corruption, while the filtered signal effectively reversed the CNR to align with the negative value observed in the motionless signals. In four out of seven aligned trials, the filtered signals achieved larger absolute CNRs, indicating their enhanced capability to detect task-related changes. The analysis in Figure 5 demonstrates the effectiveness of our method in aligning CNRs of both HbOs and HbRs with motionless signals. This alignment enhances the absolute CNR values, which are crucial for detecting task-related changes in functional near-infrared spectroscopy (fNIRS) data.

These analyses, utilizing simulated data, indicate that the 1DCNNwP surpasses existing techniques like spline interpolation, spline SG, wavelet-based methods, and TDDR. It achieves superior noise suppression and signal preservation in quasi-online filtering. This translates to more precise real-time fNIRS data processing, thereby improving the dependability of neuroimaging and brain-computer interface applications. To further validate its efficacy, an additional performance assessment using experimental data was undertaken subsequently.

**Figure 6:**
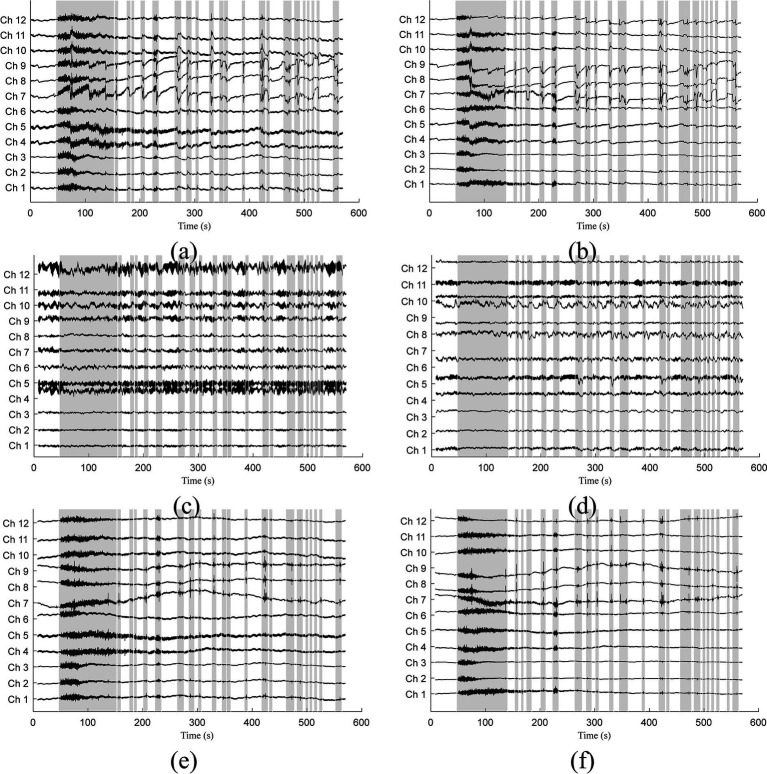
Comparison of **(A,B)** measured, **(C,D)** 1DCNNwP filtered, and **(E,F)** TDDR filtered signals (Subj. 3) for both wavelengths (*λ*_1_: left, *λ*_2_: right).

When applied to the experimental data, we used the model trained by semi-simulated data. The filtered optical and original signals for subject 3 were visually compared in Figure 6. Signals related to *λ*_1_ are displayed on the left, while those related to *λ*_2_ are on the right. Gray bars indicate sections identified as corrupted by motion artifacts. The identification process involved several steps: Downsampling the original signals by a factor of five, identifying motion artifacts using the MARA detection method, and subsequently up-sampling the artifact paradigms by five times to restore the original sampling frequency. The original signals in Figures 6A,B show the presence of both spike-like and step-like motion artifacts. Occasionally, the coexistence of these two artifact types adds complexity to the signal filtering process. Figures 6C,D displays the filtered signals after applying the 1DCNNwP method. Notably, both spike-like and step-like motion artifacts are effectively suppressed, resulting in clearer intrinsic signal patterns. Figures 6E,F illustrate the filtered signals obtained using the offline TDDR method. Upon examination, it is evident that the offline TDDR, despite its superior performance in terms of ΔSNR and ΔCC as indicated in Table 1, struggles with the effective removal of spike-like artifacts. This observation is consistent with what was seen in Figure 4C, where similar artifacts were not adequately suppressed. These results underscore that while the offline TDDR excels in enhancing signal quality as measured by ΔSNR and ΔCC, it has notable limitations when dealing with sharp, transient noise, which persist in the filtered signals. Given the brevity of the manuscript, we have only included the data from subject 3. Nevertheless, all other subjects’ figures are accessible to the general public via the link https://gitee.com/cognoholic/1-dcnnw-p.git. These results provide visual evidences of the 1DCNNwP’s capability to mitigate various types of motion artifacts while to enhance the clarity of the intrinsic signal patterns.

**Figure 7:**
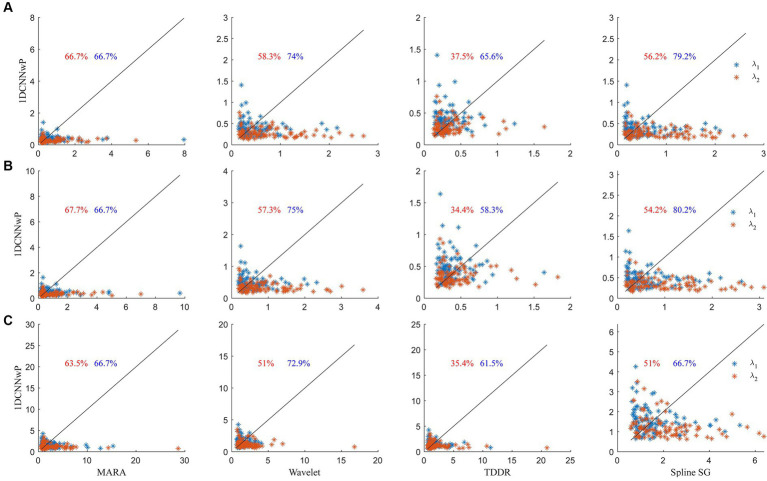
Scatter plots of **(A)** *d*_1_, **(B)** *d*_2_, and **(C)** *d*_∞_ metrics of the 1DCNNwP filter against the MARA, wavelet-based, TDDR, and spline SG filters, where the red and blue colors denote the data of *λ*_1_ and *λ*_2_, respectively; the black lines indicate the margins of the same *d*_1_, *d*_2_, and *d*_∞_ values; the numbers indicate the outperforming percentages of the 1DCNNwP filter over other filters.

The scatter plots of the proposed method vs. the wavelet-based filter, spline interpolation, and TDDR are shown in Figure 7. The performance evaluation is based on three metrics: *d*_1_, *d*_2_, and *d*_∞_. These metrics gauge the similarity between the filtered signals and reference signals obtained in the absence of head shaking, with smaller values indicating closer resemblance and thus better filter performance. In the scatter plots, each data point represents a signal set from a subject’s channel at a specific wavelength, with percentages indicating instances where 1DCNNwP metrics were superior (i.e., lower) than those of the compared filter. The analysis shows that 1DCNNwP consistently outperforms MARA, wavelet-based, and spline SG filters across all metrics for both wavelengths, indicating its efficacy in closely matching the filtered signals to the reference. For *d*_1_, 1DCNNwP shows a mixed performance against TDDR, with a notable advantage at wavelength *λ*_2_ (65.6%) but not *λ*_1_ (37.5%). In *d*_2_, indicating root-mean-squared errors, 1DCNNwP’s superiority is slightly more pronounced, particularly at *λ*_2_, though TDDR outperforms 1DCNNwP at *λ*_1_, with a reduced advantage at *λ*_2_ (58.3%). For *d*_∞_, assessing the maximum of errors, 1DCNNwP maintains its advantage over MARA, wavelet-based, and spline SG, with a similar performance gap observed with TDDR as in *d*_1_ and *d*_2_.

Statistical evaluation through left-tailed t-tests confirmed the superiority of 1DCNNwP over MARA, wavelet-based, and spline SG methods across all metrics (*d*_1_, *d*_2_, and *d*_∞_; t ranging from −7.49 to −3.82; *p* < 0.01). No significant differences were found between 1DCNNwP and TDDR for *d*_1_ and *d*_2_ metrics (*t*: 0.097 and 0.32; *p* > 0.05), yet 1DCNNwP significantly outperformed TDDR in *d*_∞_ (*t*: −2.69, *p* = 0.004 < 0.01), highlighting its effective balance in minimizing both the maximal and minimal errors in the filtered signals. These results underscore 1DCNNwP’s comprehensive capability in artifact reduction compared to other existing methods.

**Figure 8:**
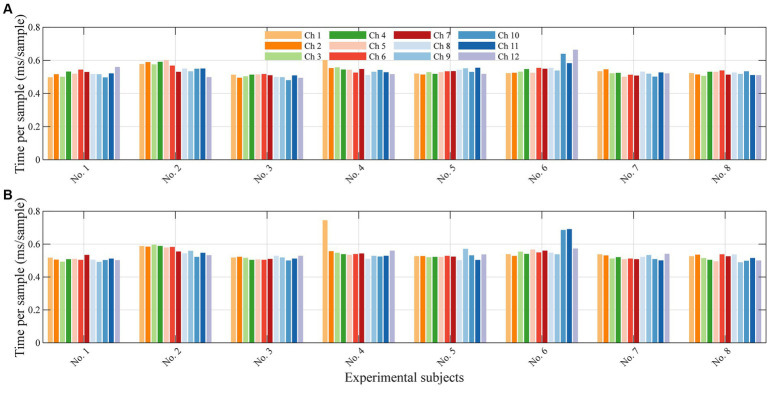
Average computational time for each sample for each subject. **(A)** shows the average time for λ_1_ and **(B)** for λ_2_.

Since the 1DCNNwP was designed for real-time processing, we also measured the filtering time consumed by the filter for each incoming new sample. The computation time consumed by the filter for each incoming new sample is depicted as bar plots in Figure 8. Each group of bars corresponds to the 12 channels, and the results are averaged across all subjects. The largest computation time recorded is 0.66 ms per sample for *λ*_1_ and 0.75 ms per sample for *λ*_2_. In contrast, the smallest computation time is 0.48 ms per sample for both wavelengths. On average, the method consumes 0.53 ms per sample. The computational time performance of the 1DCNNwP is noteworthy. It demonstrates the 1DCNNwP’s ability to handle real-time processing efficiently. For a subject, only 6.36 ms in average are required to complete the prediction of samples for all 12 channels at a given time instance. The 1DCNNwP’s computational time performance is commendable, with an average processing time of 0.53 ms per sample. This level of efficiency is crucial for real-time applications, such as brain-computer interfaces, where low-latency processing is essential. The method’s ability to handle real-time data while maintaining high noise suppression quality positions it as a valuable tool for applications. The computational efficiency was tested on an 11th Gen Intel i7-11700 with a base frequency of 2.5 GHz and 32 GB RAM. While these results demonstrate real-time capability, the algorithm’s performance on other hardware configurations has not been tested. Further investigation is needed to evaluate its scalability and efficiency across different hardware setups.

## 5 Discussion

In this study, we proposed a novel MA removal method based on a seven-layer one dimension-CNN regulated by a fully connected penalty network. The method’s development and validation comprised several critical stages. First, the neural network was meticulously trained using simulated data, laying the foundation for subsequent evaluations. The trained model was rigorously validated using simulated data, affirming its proficiency in suppressing motion artifacts and maintaining most the signal patterns. Subsequently, the model underwent further refinement through training with semi-simulated data derived from actual data. The refined training model was then validated using experimental data. Crucially, the proposed method enables real-time filtering for fNIRS data measurements, even in scenarios with limited experimental data. This is achieved through the implementation of a data-driven approach, leveraging a moving window with overlaps. The combination of these elements underscores the method’s capacity to address MA challenges in real-time fNIRS data processing, thereby advancing the field’s potential for robust and dynamic neuroimaging applications.

The proposed method was designed based on the assumption of an additive noise model. This assumption has been widely used in the existing studies (Izzetoglu et al., 2005, 2010; Robertson et al., 2010; Scholkmann et al., 2010; Huang et al., 2022), further explained using the optical model proposed (Yamada et al., 2015). Nevertheless, we distinguished the additive criteria for MAs from those for physiological noises. The physiological noises were additive in the model at the level of hemodynamic signals, whereas MAs were additive at the optical intensity level.

The evaluation of the method was designed considering three aspects: (i) ability in suppressing MAs and (ii) distortion level of signals after filtering. The overall performance was validated visually first in Figures 3, 4. Then, the MA suppression capability was evaluated using ΔSNR while the signal distortion was evaluated using ΔCC. The metrics, *d*_1_, *d*_2_, and *d*_∞_, were used to analyze experimental data, assuming that the smoothed measured signals in Session I resemble the motionless signals in Session II. The three metrics for the same signals may yield dissimilar results for different filters because filters attenuate noise differently for different norms (the absolute error, the root-mean squared error, and the maximal absolute error), as reported in some existing studies on signal processing (Chu and Delp, 1989; Huang et al., 2022). The insights gained from Figures 4, 6 provide compelling evidence of the superior performance of the proposed method in comparison to existing techniques such as MARA, the wavelet-based method, and the spline SG method (Cooper et al., 2012; Hu et al., 2015; Von Luhmann et al., 2019). The signals filtered by TDDR still suffered from positive or negative spike-like artifacts compared to our proposed method in Figure 7. These findings are reinforced by the results of the left-tailed t-test, which quantitatively confirm the method’s proficiency in mitigating extremum in errors (*d*_∞_) regarding the proposed method and TDDR. Still, one cannot conclude any significance in the absolute errors (*d*_1_) and root-mean-squared errors (*d*_2_) to them. In fact, for all three evaluation metrics (*d*_1_, *d*_2_, and *d*_∞_), the proposed method demonstrates significant advantages over the other techniques, with *p*-values consistently below 0.01. This statistical evidence further underscores the method’s effectiveness in motion artifact attenuation. It is important to note that while the advantages may appear subtle in certain scenarios (e.g., for *λ*_1_ in the sense of *d*_1_), the collective results emphasize the substantial leap in performance offered by our approach over conventional methods.

The practical application of the 1DCNNwP in real-time scenarios hinges on the timely processing of neural data. In this context, signal processing time becomes a critical factor. In our study, we meticulously evaluated and reported the average signal processing time for each new sample in every channel. However, it is essential to consider that in many commercial fNIRS devices, optical signals are acquired sequentially, or in series within groups, positioned in proximity to prevent signal coupling among closely situated light sources. As a result, the actual sampling time should align with multiples of the number of channels within these groups, which we refer to as cycling time. Our findings reveal that the average signal processing time for a single wavelength in a single channel is 0.53 ms. Consequently, when applied to 12 channels, the total processing time per cycle amounts to 12.72 ms for both wavelengths. Despite having 2.1 million parameters, the complexity of 1DCNNwP does not significantly increase latency in data processing. The *O*(*n*^2^) complexity of fully connected layers in the penalty branch is manageable since *n* refers to the number of neurons (2 *W*). The computational time analysis supports this conclusion.

The acceptability of this timing for real-time processing in fNIRS-based brain-computer interfaces hinges on several factors. It is notably dependent on the specific requirements and constraints of the application. In some contexts, a cycle time of 12.72 ms may be acceptable, while in others, it may introduce perceptible delays. The suitability of this timing should be evaluated in the context of the particular application’s latency tolerance. Moreover, the method’s demonstrated effectiveness in motion artifact suppression and real-time capabilities may outweigh the modest processing time in scenarios where real-time processing is paramount. Unlike the conventional.

Finally, the proposed algorithm provides a possible online filtering solution for the motion-corrupted fNIRS data. Nevertheless, two limitations of the algorithm are that (i) the current model is signal-specific, designed to fit the data from the channel for which it was originally trained, and (ii) the proposed method ignored the multivariate effect of MAs that involve multi-channels. The first limit implies that the training process for each new subject is inevitable. This guarantees a subject specific MA removal solution but also adds to the complexity in the preparation phase. As for the second constraint, incorporating multivariant verification can enhance the accuracy of MA identification. However, such an incorporation can cause new challenges in multivariant fNIRS data simulations. In light of the two constraints, future research will concentrate on assessing the method’s applicability across various experimental scenarios and its adaptability among different subjects. This focus may substantially enhance the algorithm’s generalization to practical applications.

## 6 Conclusion

In this paper, we introduced a novel approach designed to facilitate real-time motion artifact (MA) removal through a subject-specific approach. The proposed method is rooted in a seven-layer one-dimensional convolutional neural network intricately governed by a fully connected penalty network. The validation of this approach involved comprehensive assessments using both simulated and experimental data, executed in a pseudo-real-time processing fashion. Simulation validation results underscored the method’s outstanding performance, demonstrating that 1DCNNwP surpassed or exhibited comparable efficacy to established techniques like the spline-interpolation method (MARA), the wavelet-based method, the temporal derivative distribution repair (TDDR) with a 1-s moving window, and the spline Savitzky–Golay (spline SG) method in both noise suppression (ΔSNR) and signal distortion (ΔCC). Substantiating these simulation outcomes, experimental results further established the method’s superiority over these methods (except for offline TDDR) with a statistically significant advantage, as confirmed by the critical *p*-value threshold of 0.01 in *t* tests. The achievement of an average processing time of 0.53 ms per sample further reinforces the method’s applicability in real-time scenarios. In summary, our proposed approach advances the field of MA removal in fNIRS, offering a robust, efficient, and subject-specific solution with the potential to enhance the quality of neuroimaging and brain-computer interface applications.

## Data availability statement

The raw data supporting the conclusions of this article will be made available by the authors, without undue reservation.

## Ethics statement

The studies involving humans were approved by Institutional Review Board of Shenzhen Institute of Advanced Technology, Chinese Academy of Sciences. The studies were conducted in accordance with the local legislation and institutional requirements. The participants provided their written informed consent to participate in this study.

## Author contributions

RH: Writing – review & editing, Writing – original draft, Formal analysis, Data curation. K-SH: Writing – review & editing, Supervision, Resources, Conceptualization. S-CB: Writing – review & editing, Validation, Investigation. FG: Writing – review & editing, Validation, Supervision, Methodology.
